# A multi-national, randomised, open-label, parallel, phase III non-inferiority study comparing NK105 and paclitaxel in metastatic or recurrent breast cancer patients

**DOI:** 10.1038/s41416-019-0391-z

**Published:** 2019-02-12

**Authors:** Yasuhiro Fujiwara, Hirofumi Mukai, Toshiaki Saeki, Jungsil Ro, Yung-Chang Lin, Shigenori E. Nagai, Keun Seok Lee, Junichiro Watanabe, Shoichiro Ohtani, Sung Bae Kim, Katsumasa Kuroi, Koichiro Tsugawa, Yutaka Tokuda, Hiroji Iwata, Yeon Hee Park, Youngsen Yang, Yoshihiro Nambu

**Affiliations:** 1 Department of Breast and Medical Oncology, National Cancer Center Hospital, Tokyo, Japan; 2 Division of Breast and Medical Oncology, National Cancer Center Hospital East, Chiba, Japan; 3 Department of Breast Oncology, Saitama Medical University International Medical Center, Saitama, Japan; 4 Graduate School of Cancer Science and Policy, National Cancer Center, Goyang, Korea; 5 Division of Haematology and Oncology, Chang-Gung Memorial Hospital, Linko, Taoyuan, Taiwan; 6 Division of Breast Oncology, Saitama Cancer Center, Saitama, Japan; 7 Center for Breast Cancer, National Cancer Center, Goyang, Korea; 8 Division of Breast Oncology, Shizuoka Cancer Center, Shizuoka, Japan; 9 Department of Breast Surgery, Hiroshima City Hiroshima Citizens Hospital, Hiroshima, Japan; 10 Department of Oncology, Asan Medical Center, University of Ulsan College of Medicine, Seoul, Korea; 11 Department of Surgery, Tokyo Metropolitan Cancer and Infectious Diseases Center Komagome Hospital, Tokyo, Japan; 12 Division of Breast and Endocrine Surgery, Department of Surgery, St. Marianna University School of Medicine, Kawasaki, Japan; 13 Department of Breast and Endocrine Surgery, Tokai University School of Medicine, Isehara, Japan; 14 Department of Breast Oncology, Aichi Cancer Center Hospital, Nagoya, Japan; 15 Division of Hematology-Oncology, Samsung Medical Center, Seoul, Korea; 16 Division of Hematology-Oncology, Department of Internal Medicine, Taichung Veterans General Hospital, Taichung, Taiwan; 17 Internal Medicine, College of Medicine, China Medical University, Taichung, Taiwan; 18 Pharmaceuticals Group, Nippon Kayaku Co., Ltd., Tokyo, Japan

**Keywords:** Breast cancer, Breast cancer, Breast cancer, Breast cancer

## Abstract

**Background:**

NK105 is a novel nanoparticle drug delivery formulation that encapsulates paclitaxel (PTX) in polymeric micelles. We conducted an open-label phase III non-inferiority trial to compare the efficacy and safety of NK105 and PTX in metastatic or recurrent breast cancer.

**Methods:**

Patients were randomly assigned in a 1:1 ratio to receive either NK105 (65 mg/m^2^) or PTX (80 mg/m^2^) on days 1, 8 and 15 of a 28-day cycle. The primary endpoint was progression-free survival (PFS), with a non-inferiority margin of 1.215.

**Results:**

A total of 436 patients were randomised and 211 patients in each group were included in the efficacy analysis. The median PFS was 8.4 and 8.5 months for NK105 and PTX, respectively (adjusted hazard ratio: 1.255; 95% confidence interval: 0.989–1.592). The median overall survival and overall response rates were 31.2 vs. 36.2 months and 31.6% vs. 39.0%, respectively. The two groups exhibited similar safety profiles. The incidence of peripheral sensory neuropathy (PSN) was 1.4% vs. 7.5% (≥Grade 3) for NK105 and PTX, respectively. The patient-reported outcomes of PSN were significantly favourable for NK105 (*P* < 0.0001).

**Conclusions:**

The primary endpoint was not met, but NK105 had a better PSN toxicity profile than PTX.

**Clinical trial registration:**

ClinicalTrials.gov: NCT01644890

## Introduction

Breast cancer is the second most common cancer in the world and the fifth most common cause of cancer death.^1^ Although the last two decades have witnessed extraordinary progress in the understanding of the disease and in improved outcomes for women with early-stage disease, recurrence or metastases remain largely incurable.

Paclitaxel (PTX) is a key drug in breast cancer treatment but has some clinical issues associated with potential hypersensitivity to its vehicle, polyoxyethylene castor oil or ethanol. Steroid and/or antihistamine pre-treatment must therefore be administered to patients receiving PTX to prevent serious hypersensitivity caused by polyoxyethylene castor oil. Moreover, although long-term treatment is useful for advanced breast cancer, PTX-induced peripheral sensory neuropathy (PSN) often interferes with continuous PTX treatment.

NK105 is a PTX-incorporating “core-shell-type” polymeric micellar nanoparticle formulation that can be administered intravenously without polyoxyethylene castor oil or ethanol.

A polymeric micellar such as NK105 might have a notable “enhanced permeability and retention (EPR) effect”, and our past non-clinical study showed that NK105 affords efficacy superior to that of PTX, the efficacy of which is attributed to this effect.^2^ The EPR effect is a unique phenomenon that arises because of the pathophysiological characteristics of solid tumour tissue: hypervascularity, incomplete vascular architecture, secretion of vascular permeability factors stimulating extravasation within cancer tissue and absence of effective lymphatic drainage from tumours, which prevents the efficient clearance of macromolecules accumulated in solid tumours.^3,4^

Our two clinical studies showed that NK105 affords good efficacy which might be attributed to the EPR effect. In a phase I study in solid tumours and breast cancer, all patients who received three or more treatment cycles (*n* = 7) achieved partial response or stable disease in the dose-escalation cohort (*n* = 15), and the overall response rate in the following expansion cohort (*n* = 10) was 60%.^5^ In a phase II study evaluating a tri-weekly regimen of NK105 in treated advanced gastric cancer, NK105 (150 mg/m^2^) had a preferable response rate of 25.0% (*n* = 56), including 2 complete responses.^6^ These results suggested the potential of the EPR effect on the mode of action for NK105.

The initial dose of NK105 in this study was 65 mg/m^2^, which was selected taking into consideration the results of a phase I study in solid tumours. In this phase I study, 80 mg/m^2^ had been selected as the recommended weekly dose of NK105. However, in the following expansion phase, in which NK105 was administered at a dose of 80 mg/m^2^, neutropenia was frequently observed, and treatment often had to be postponed or the dose reduced. NK105 was expected to afford comparable efficacy to PTX even though the dose of NK105 was lower than that of PTX because of the EPR effect, as shown in the previous studies. Based on these results, 65 mg/m^2^ was ultimately selected as the dose for this study.^5^

This study therefore aimed to verify the non-inferiority of NK105 (65 mg/m^2^) to PTX (80 mg/m^2^) based on progression-free survival (PFS) in metastatic or recurrent breast cancer and to compare their safety profiles especially focussed on the cumulative incidence and patient-reported outcomes of PSN.

## Patients and methods

### Patients

The key inclusion criteria included female sex; age 20 to 74 years at the time of informed consent; histologically confirmed metastatic or recurrent adenocarcinoma of the breast; presence of a measurable lesion according to the Response Evaluation Criteria in Solid Tumours (RECIST) version 1.1; and Eastern Cooperative Oncology Group performance status ≤ 1.

The key exclusion criteria included recurrence within 1 year after the last dose of a neoadjuvant and/or adjuvant taxane; prior systemic taxane-based chemotherapy for metastatic or recurrent breast cancer; prior systemic chemotherapy with two or more regimens for metastatic or recurrent breast cancer; eligibility for anti-HER2 therapy; presence of grade 2 or greater PSN or grade 1 or greater PSN in the presence of diabetes at randomisation.

Written informed consent was obtained from each patient prior to entry into the study. The study was registered in ClinicalTrials.gov with the number NCT01644890.

### Study design

This study was a multi-national, randomised, open-label, parallel, phase III non-inferiority study. Patients were randomly assigned in a 1:1 ratio to receive either NK105 or PTX using an interactive web response system. Randomisation was performed centrally, with minimisation stratified by history of chemotherapy for metastatic or recurrent breast cancer, history of treatment with a taxane, oestrogen receptor status, disease-free interval and site.

### Treatment

Patients received either NK105 (65 mg/m^2^) or PTX (80 mg/m^2^) on days 1, 8 and 15 of a 28-day cycle. The prepared solution of NK105 was infused over a period of about 30 min. The prepared solution of PTX was infused over a period of about 1 h. Patients who received PTX also received prophylactic premedication with an antihistamine, corticosteroid and/or H2 receptor antagonist to prevent hypersensitivity. Study treatment was discontinued when disease progression occurred, as determined on the basis of clinical findings or image assessment in accordance with RECIST Ver. 1.1; an adverse event occurred which led investigators to conclude that continuing the study would be difficult; or when the patient requested discontinuation. Measures taken in response to ≥grade 3 non-haematological toxicities associated with study treatment and ≥grade 2 neutropenia and/or thrombocytopenia included skipping doses, reducing the dose and the administration of symptomatic therapy. The use of granulocyte-colony stimulating factor (G-CSF) in accordance with the American Society of Clinical Oncology Guideline^7^ recommendations was permitted during the study. Patients meeting the criteria for study treatment discontinuation were transitioned to the post-treatment observation period and follow-up investigations of their survival were performed every 3 months.

### Evaluations

The primary endpoint was PFS, defined as the period from the day of randomisation until the first observation of lesion progression or death from any cause. Key secondary end points for efficacy included overall survival (OS) and overall response rate (ORR). Tumour assessments were performed every 6 weeks until disease progression according to RECIST version 1.1. Assessment of antitumour efficacy was evaluated by a blinded independent central review laboratory and the investigator, and the former was used for the primary analysis.

Adverse events were graded using the Common Terminology Criteria for Adverse Events version 4.03 and were classified using the Medical Dictionary for Regulatory Activities version 19.0. The cumulative incidences of PSN were estimated by time to onset of PSN. Patient-reported outcomes of PSN were assessed on day 1 of each cycle prior to administration and medical examination using the “additional concerns” subscale of FACT/GOG-NTX version 4 (FACT/GOG-NTX subscale). An independent data and safety monitoring board supervised the conduct of the study and regularly assessed the safety profile.

### Statistical analysis

Based on the results of previous studies for paclitaxel in breast cancer, the expected median PFS was 5.5 months for PTX and 6.35 months for NK105. Then, assuming a randomisation period of 18 months, a follow-up period of 12 months, a one-sided significance level of 2.5%, a power of 85% and the above-described non-inferiority margin of 1.215, it was estimated that the number of patients necessary to verify the non-inferiority of NK105 to PTX would be 172 patients per group, a total of 344 patients.^8–15^ Considering an expected withdrawal/dropout rate of approximately 20%, the target sample size was set at 414. The full analysis set (FAS), consisting of all randomised patients who received study drug at least once and who had no major violations of the eligibility criteria, was used for the efficacy analysis. The primary PFS analysis was confirmed whether or not the upper limit of the 95% confidence interval (CI) for the hazard ratio (HR) for NK105 relative to PTX fell below the non-inferiority margin of 1.215 (<1.215) by fitting a Cox proportional hazards model that included allocation adjustment factors other than the study site as covariates. The same Cox proportional hazards model was used to derive the HR for OS with a 95% CI. The Kaplan–Meier method was used to estimate the PFS and OS curves. The median PFS and median OS and 95% CIs thereof were calculated. The ORR was calculated along with the 95% CIs for each group.

Safety data were summarised descriptively using the safety analysis set, which comprised all randomised patients who received study drug at least once. Furthermore, the time to PSN onset was reported as the cumulative incidence of PSN using the reverse Kaplan–Meier method and the median time to PSN onset was calculated along with the 95% CI thereof for each group. The time to PSN onset was compared using a log-rank test. For the FACT/GOG-NTX subscale, a repeated-measures analysis of variance in FAS with at least one post-treatment assessment was carried out using group and time point as fixed effects, patient’s effect as the random effect and the baseline value as a covariate. Statistical analyses were performed using SAS software (version 9.4; SAS Institute, Cary, NC).

## Results

### Patients

From September 2012 to July 2014, a total of 436 female patients were randomly assigned centrally in a 1:1 ratio to either NK105 or PTX from 58 sites in Japan (27 sites), Korea (19 sites) and Taiwan (12 sites). Four patients in the NK105 group and five patients in the PTX group were excluded from the safety analysis after randomisation. Therefore, a total of 427 patients were included in the safety analysis. Furthermore, three patients in the NK105 group and two patients in the PTX group were excluded from the efficacy analysis after study drug administration. Thus, a total of 422 patients were ultimately included in the efficacy analysis (Supplementary Figure S1). The main baseline characteristics of the patients were well balanced between groups (Table 1).

**Table 1:** Baseline patient characteristics

Characteristic	NK105 (*N* = 211)	PTX (*N* = 211)	Total (*N* = 422)
Age (year)			
Mean ± SD	55.3 ± 10.2	55.0 ± 10.6	55.2 ± 10.4
Median	56.0	56.0	56.0
Min, max	29, 73	24, 74	24, 74
Enroled country, *n* (%)			
Japan	136 (64.5)	142 (67.3)	278 (65.9)
Korea	50 (23.7)	41 (19.4)	91 (21.6)
Taiwan	25 (11.8)	28 (13.3)	53 (12.6)
ECOG performance status			
0	145 (68.7)	152 (72.0)	297 (70.4)
1	66 (31.3)	59 (28.0)	125 (29.6)
Oestrogen receptor, *n* (%)			
Positive	167 (79.1)	169 (80.1)	336 (79.6)
Negative	43 (20.4)	42 (19.9)	85 (20.1)
Unknown	1 (0.5)	0	1 (0.2)
Progesteron receptor, *n* (%)			
Positive	123 (58.3)	137 (64.9)	260 (61.6)
Negative	86 (40.8)	73 (34.6)	159 (37.7)
Unknown	2 (0.9)	1 (0.5)	3 (0.7)
Triple-negative, *n* (%)			
Yes	37 (17.5)	40 (19.0)	77 (18.2)
No	174 (82.5)	171 (81.0)	345 (81.8)
History of treatment using a taxane, *n* (%)			
Yes	64 (30.3)	65 (30.8)	129 (30.6)
No	147 (69.7)	146 (69.2)	293 (69.4)
Prior chemotherapy			
Neoadjuvant	36 (17.1)	40 (19.0)	76 (18.0)
Adjuvant	80 (37.9)	77 (36.5)	157 (37.2)
Recurrent	28 (13.3)	24 (11.4)	52 (12.3)
Metastatic	22 (10.4)	26 (12.3)	48 (11.4)
Other	2 (0.9)	0 (0.0)	2 (0.5)
None	86 (40.8)	80 (37.9)	166 (39.3)
Breast cancer, *n* (%)			
Primary	71 (33.6)	75 (35.5)	146 (34.6)
Recurrent	140 (66.4)	136 (64.5)	276 (65.4)
Disease-free interval (months), *n* (%)			
<12 (Including newly metastatic disease)	82 (38.9)	83 (39.3)	165 (39.1)
≥12	129 (61.1)	128 (60.7)	257 (60.9)

*PTX* paclitaxel, *ECOG* Eastern Cooperative Oncology Group

The median duration of treatment of NK105 and PTX was 6.5 (1–36) and 7.4 months (1–37), respectively. The median dose intensity of NK105 and PTX was 44.76 mg/m^2^/week and 54.33 mg/m^2^/week, respectively. The median relative dose intensity of NK105 and PTX was 94.53% and 92.39%, respectively.

### Efficacy

**Fig. 1:**
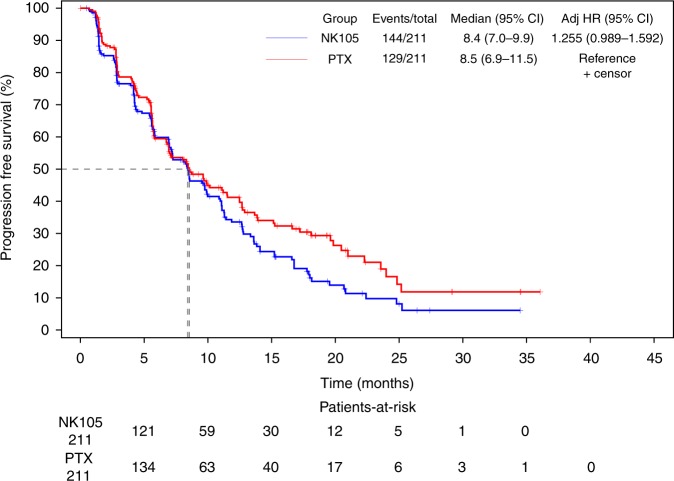
Kaplan–Meier curves for progression-free survival in full analysis set

**Fig. 2:**
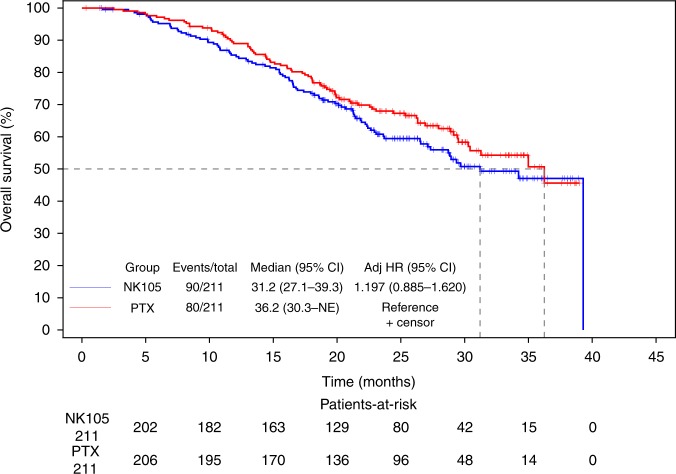
Kaplan–Meier curves for overall survival in full analysis set

The median PFS of NK105 and PTX was 8.4 months (95% CI: 7.0–9.9) and 8.5 months (95% CI: 6.9–11.5), respectively (adjusted HR: 1.255; 95% CI: 0.989–1.592; Fig. 1), which exceeded the predefined non-inferiority margin of 1.215. The median OS of NK105 and PTX was 31.2 months (95% CI: 27.1–39.3) and 36.2 months (95% CI: 30.3–NE), respectively (adjusted HR: 1.197; 95% CI: 0.885–1.620; Fig. 2).

The ORR of NK105 and PTX was 31.6% and 39.0%, respectively (Supplementary Table S1). Also, the difference in ORR between treatment groups was −7.5%.

### Safety and patient-reported outcomes

**Fig. 3:**
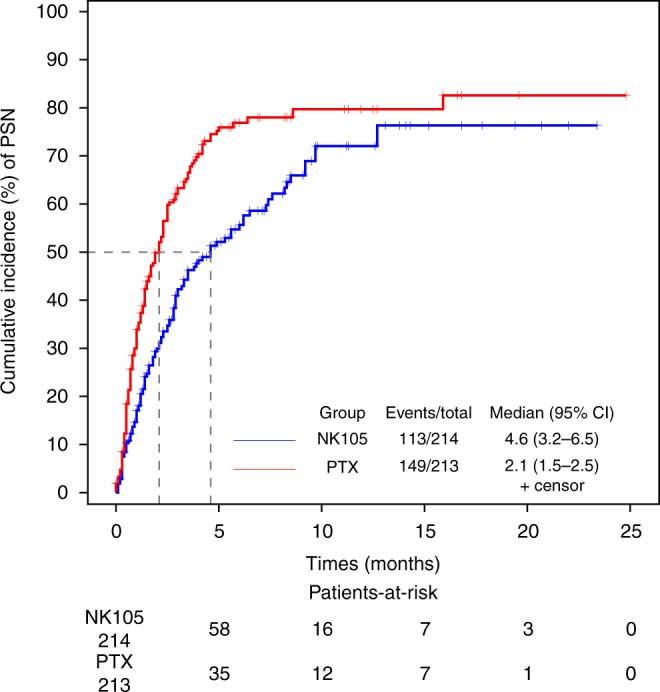
Cumulative incidence of peripheral sensory neuropathy

**Fig. 4:**
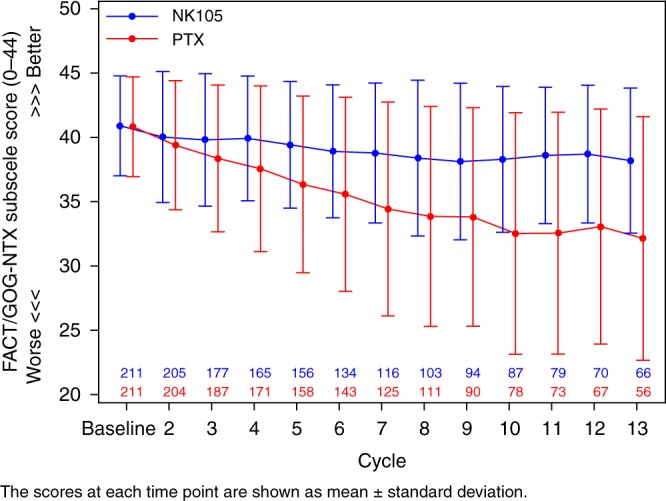
Patient-reported outcomes of peripheral sensory neuropathy assessed by FACT/GOG-NTX subscale

In the safety analysis, 7 (3.3%) of 214 patients in the NK105 group and 23 (10.8%) of 213 patients in the PTX group discontinued treatment due to adverse events. All grade adverse events occurring in 20% or more of the patients in either group were reported in Table 2. The most common adverse events in both groups were alopecia (70.6% vs. 75.6%) and PSN (52.8% vs. 70.0%). The most common grade 3 or higher adverse events in both groups were neutropenia (33.6% vs. 30.5%) and leukopenia (15.9% vs. 14.1%). The safety profile was similar in both groups. Nine patients (4.2%) in the NK105 group and 11 patients (5.2%) in the PTX group used G-CSF at least once. There were three treatment-related deaths in the PTX group, compared with none in the NK105 group. The incidence of grade 1 PSN was similar in both groups (41.1% vs. 41.3%), and the incidences of grade 2 and grade 3 PSN were better in the NK105 group (10.3% vs. 21.1% and 1.4% vs. 7.5%, respectively). No grade 4 PSN occurred in either group. The resolution of PSN in the NK105 group was also favourable, and the rates of the patients whose PSN status was “persistent” at the end of safety assessment were 80.5% (91/113) in the NK105 group and 89.3% (133/149) in the PTX group. The cumulative incidence of PSN was significantly lower in the NK105 group (*P* < 0.0001) (Fig. 3). Furthermore, patient-reported outcomes of PSN were significantly more favourable in the NK105 group *(P* < 0.0001) (Fig. 4). Patients with grade 1 PSN were allowed to enrol in this study if they did not have diabetes, and these patients were well balanced between the two groups (5.6% vs. 6.5%). A total of 46 patients (21.5%) in the NK105 group and all patients in the PTX group received some kind of premedication. Hypersensitivities were reported in 9 patients (4.2%) in the NK105 group and 10 patients (4.7%) in the PTX group.

**Table 2:** Adverse events occurring in 20% or more patients in either group

Adverse events	NK105 (*N* = 214)			PTX (*N* = 213)		
	Grade			Grade		
Preferred term	Any	3	4	Any	3	4
Haematological, *n* (%)						
Neutropenia^a^	109 (50.9)	54 (25.2)	18 (8.4)	103 (48.4)	55 (25.8)	10 (4.7)
Leukopenia^b^	72 (33.6)	32 (15.0)	2 (0.9)	68 (31.9)	30 (14.1)	0 (0.0)
Non-haematological, *n* (%)						
Alopecia	151 (70.6)	0 (0.0)	0 (0.0)	161 (75.6)	0 (0.0)	0 (0.0)
Peripheral sensory neuropathy	113 (52.8)	3 (1.4)	0 (0.0)	149 (70.0)	16 (7.5)	0 (0.0)
Rash	62 (29.0)	0 (0.0)	0 (0.0)	47 (22.1)	0 (0.0)	0 (0.0)
Nausea	59 (27.6)	5 (2.3)	0 (0.0)	65 (30.5)	0 (0.0)	0 (0.0)
Nasopharyngitis	49 (22.9)	0 (0.0)	0 (0.0)	46 (21.6)	0 (0.0)	0 (0.0)
Diarrhoea	47 (22.0)	4 (1.9)	0 (0.0)	41 (19.2)	1 (0.5)	0 (0.0)
Fatigue	45 (21.0)	1 (0.5)	0 (0.0)	36 (16.9)	0 (0.0)	0 (0.0)
Stomatitis	42 (19.6)	2 (0.9)	0 (0.0)	46 (21.6)	0 (0.0)	0 (0.0)
Nail discolouration	37 (17.3)	0 (0.0)	0 (0.0)	46 (21.6)	0 (0.0)	0 (0.0)
Myalgia	32 (15.0)	0 (0.0)	0 (0.0)	46 (21.6)	0 (0.0)	0 (0.0)
Dysgeusia	30 (14.0)	0 (0.0)	0 (0.0)	53 (24.9)	0 (0.0)	0 (0.0)

^a^Neutropenia included neutrophil count decreased

^b^Leukopenia included white blood cell count decreased

## Discussion

This study aimed to verify the non-inferiority of NK105 to PTX based on the PFS, a standard therapy for metastatic or recurrent breast cancer, and was the first comparison study in which an EPR effect was expected. Based on the results of the phase I trial, NK105 65 mg/m^2^, one dose level lower than the recommended dose (80 mg/m^2^), and a weekly PTX dose of 80 mg/m^2^, the commonly used dose, were selected as the initial dose levels in this study.

This study did not demonstrate the non-inferiority in PFS of NK105 compared to PTX (8.4 months (95% CI: 7.0–9.9) vs. 8.5 months (95% CI: 6.9–11.5); adjusted HR: 1.255 (95% CI: 0.989–1.592)). Although NK105 was expected to afford comparable or superior efficacy to PTX due to the EPR effects that had been reported in a non-clinical study,^2^ the contribution of the EPR effect was limited in this study. In view of the limited EPR effect, the lower dose intensity of NK105 could be one of the reasons for not being able to accomplish the primary endpoint.

According to several recent studies evaluating weekly single doses of PTX (80–90 mg/m^2^) in breast cancer, PTX monotherapy affords a PFS of 6.89 months (3.38–8.8),^12,16–18^ an OS of 20.99 months (10.35–25.2),^9–12,16–18^ and an ORR of 26.73% (21.1–43.5).^9–12,15–18^ In this study, although the dose of NK105 was lower than that of PTX, there was not much difference between the two in the PFS, OS or ORR (median PFS: 8.4 months vs. 8.5 months; median OS: 31.2 months vs. 36.2 months; mean ORR: 31.6% vs. 39.8%).

In this study, the median PFS with PTX was longer than expected, although no clear reason for this difference was found.

The relative dose intensity was similar in both groups (94.52% vs. 92.39%), and treatment compliance was therefore good, and NK105 65 mg/m^2^ was well tolerated in long-term use. The incidence of treatment-related discontinuations was only 3.3% in the NK105 group compared to 10.8% in the PTX group.

The profile of haematological toxicity was similar in both groups, but the profile of non-haematological toxicity was better in the NK105 group than in the PTX group. NK105 had a better PSN profile and a lower incidence of high-grade PSN than PTX. The patient-reported outcomes of PSN were slightly lower in the NK105 group, and no major changes were found throughout the study period, although the PTX score decreased over the course of time.

Although no premedication was mandatory for NK105 treatment, 21.5% patients received some kind of premedication in the first administration. After seven cycles, no patients in the NK105 group received any premedication. The NK105 infusion time was only 30 min, against 60 min for PTX. No severe hypersensitivity or infusion-related reactions occurred in either group, which showed the validity of the dosing method for NK105 in this study.

The haematological toxicity profile of NK105 was similar to that of PTX. Only a few grade 3 or higher infections occurred in both groups (3.2% vs. 4.2%). Febrile neutropenia was reported in only one patient in each group, and both were grade 3. Also, no severe thrombocytopenia was observed.

In conclusion, while this study did not demonstrate the non-inferiority of NK105 to PTX based on the PFS, overall efficacy measurements did not differ much between the two groups. The safety profile of NK105 was well tolerated and the PSN profile of NK105 was particularly favourable in comparison with that of PTX. The efficacy of NK105 should be re-evaluated in future studies.

## Supplementary information

Supplementary Figure S1

Supplementary Table S1

Supplementary Table S2

## **Data availability:**

The data generated and analysed during this study are available at ClinicalTrials.gov (NCT01644890).
