# Supplementary material for: A multi-national, randomised, open-label, parallel, phase III non-inferiority study comparing NK105 and paclitaxel in metastatic or recurrent breast cancer patients
Source: Br J Cancer. 2019 Feb 12;120(5):475–80. doi: 10.1038/s41416-019-0391-z (PMC6461876; doi:10.1038/s41416-019-0391-z)
Supplement: Supplementary file 1 — Supplementary Figure S1 [file 41416_2019_391_MOESM1_ESM.docx]

Supplementary Figure S1. Consort flow diagram

Exclude from per protocol set (*n* = 0)

Exclude from per protocol set (*n* = 1)

Protocol deviation (*n* = 1)

Exclude from full analysis set (*n* = 2)

Ineligible after intervention (*n* = 2)

Exclude from full analysis set (*n* = 3)

Ineligible after intervention (*n* = 3)

Safety analysis set (*n* = 213)

Safety analysis set (*n* = 214)

Did not receive NK105 (*n* = 4)

Ineligible (*n* = 3)

Adverse event (*n* = 1)

Allocated to NK105 (*n* = 218)

Enrolled and randomized (*n* = 436)

Did not receive PTX (*n* = 5)

Ineligible (*n* = 2)

Protocol deviation (*n* = 1)

Alcohol intolerance (*n* = 1)

Withdrawal (*n* = 1)

Per protocol set (*n* = 211)

Per protocol set (*n* = 210)

Full analysis set (*n* = 211)

Full analysis set (*n* = 211)

## Analysis

Allocated to PTX (*n* = 218)

## Allocation
