# Supplementary material for: A multi-national, randomised, open-label, parallel, phase III non-inferiority study comparing NK105 and paclitaxel in metastatic or recurrent breast cancer patients
Source: Br J Cancer. 2019 Feb 12;120(5):475–80. doi: 10.1038/s41416-019-0391-z (PMC6461876; doi:10.1038/s41416-019-0391-z)
Supplement: Supplementary file 2 — Supplementary Table S1 [file 41416_2019_391_MOESM2_ESM.docx]

Supplementary Table S1. Response rate for the patients with target lesion in full analysis set

| Item | Classification | NK105 (*N* = 211) | PTX (*N* = 211) |
| --- | --- | --- | --- |
| Patients with target lesion, *n* (%) | Yes | 190 (90.0%) | 187 (88.6%) |
|  | No | 21 (10.0%) | 24 (11.4%) |
| Best overall response, *n* (%) | CR^b^ | 0 (0.0%) | 1 (0.5%) |
|  | PR^c^ | 60 (31.6%) | 72 (38.5%) |
|  | SD^d^ | 95 (50.0%) | 86 (46.0%) |
|  | PD^e^ | 31 (16.3%) | 23 (12.3%) |
|  | NE^f^ | 4 (2.1%) | 5 (2.7%) |
| ORR^a^ | Number of CR+PR | 60 | 73 |
|  | ORR | 31.6% | 39.0% |
|  | 95%CI^g^ | (25.0%, 38.7%) | (32.0%, 46.4%) |
|  | Difference in ORR | -7.5% | |
|  | 95%CI | (-17.4%, 2.7%) | |
| ^a^overall response rate ^b^complete response ^c^partial response  ^d^stable disease ^e^progressive disease ^f^not evaluable ^g^95% confidence interval | | | |
