# Supplementary material for: A multi-national, randomised, open-label, parallel, phase III non-inferiority study comparing NK105 and paclitaxel in metastatic or recurrent breast cancer patients
Source: Br J Cancer. 2019 Feb 12;120(5):475–80. doi: 10.1038/s41416-019-0391-z (PMC6461876; doi:10.1038/s41416-019-0391-z)
Supplement: Supplementary file 3 — Supplementary Table S2 [file 41416_2019_391_MOESM3_ESM.docx]

Supplementary Table S2. Institution list

| **Country** | **Institution** | **Investigator** |
| --- | --- | --- |
| Japan | National Hospital Organization Hokkaido Cancer Center | Masato TAKAHASHI |
| Japan | Iwate Medical University Hospital | Kazushige ISHIDA |
| Japan | Saitama Medical University International Medical Center | Toshiaki SAEKI |
| Japan | Saitama Cancer Center | Shigenori NAGAI |
| Japan | National Cancer Center Hospital East | Hirofumi MUKAI |
| Japan | National Cancer Center Hospital | Yasuhiro FUJIWARA |
| Japan | St Luke's International Hospital | Teruo YAMAUCHI |
| Japan | Showa University Hospital | Takashi KUWAYAMA |
| Japan | Shizuoka Cancer Center | Junichiro WATANABE |
| Japan | Hiroshima City Hiroshima Citizens Hospital | Shoichiro OHTANI |
| Japan | Sagara Hospital | Yoshiaki RAI |
| Japan | Tohoku University Hospital | Takanori ISHIDA |
| Japan | Juntendo University Hospital | Mitsue SAITO |
| Japan | Komagome Hospital | Katsumasa KUROI |
| Japan | Tokai University School of Medicine | Yutaka TOKUDA |
| Japan | St. Marianna University School of Medicine Hospital | Koichiro TSUGAWA |
| Japan | Aichi Cancer Center Hospital | Hiroji IWATA |
| Japan | National Hospital Organization Osaka National Hospital | Norikazu MASUDA |
| Japan | Kitakyushu Municipal Medical Center | Keisei ANAN |
| Japan | Kurume General Hospital | Miki YAMAGUCHI |
| Japan | Osaka University Hospital | Atsushi SHIMOMURA |
| Japan | Chiba Cancer Center | Rikiya NAKAMURA |
| Japan | Kanagawa Cancer Center | Satoru SHIMIZU |
| Japan | Nippon Medical School Musashi Kosugi Hospital | Noriyuki KATSUMATA |
| Japan | National Hospital Organization Kyushu Cancer Center | Eriko TOKUNAGA |
| Japan | National Hospital Organization Shikoku Cancer Center | Shozo OHSUMI |
| Japan | Jichi Medical University Hospital | Takashi FUJITA |
| Korea | National Cancer Center | Keun Seok LEE |
| Korea | Severance Hospital, Yonsei University Health System | Joo Hyuk SOHN |
| Korea | Asan Medical Center | Sung-Bae KIM |
| Korea | Dong-A University Hospital | Suee LEE |
| Korea | Korea University Anam Hospital | Yoon Ji CHOI |
| Korea | Seoul National University Bundang Hospital | Jee Hyun KIM |
| Korea | Ulsan University Hospital | Su-Jin KOH |
| Korea | Pusan National University Hospital | Joo Seop CHUNG |
| Korea | Keimyung University Dongsan Hospital | Hong Suk SONG |
| Korea | Kyungpook National University Medical Center | Yee Soo CHAE |
| Korea | Seoul National University Hospital | Seock-Ah IM |
| Korea | Samsung Medical Center | Yeon Hee PARK |
| Korea | Chungbuk National University Hospital | Ki Hyeong LEE |
| Korea | Korea University Guro Hospital | Jae Hong SEO |
| Korea | Gangnam Severance Hospital, Yonsei University Health System | Joon JEONG |
| Korea | Korea Cancer Center Hospital, Korea Institute of Radiological & medical sciences | Woo Chul NOH |
| Taiwan | Chang Gung Memorial Hospital, Linkou | Yung-Chang LIN |
| Taiwan | National Cheng Kung University Hospital | Wu-Chou SU |
| Taiwan | Mackay Memorial Hospital | Yuan-Ching CHANG |
| Taiwan | Taipei Veterans General Hospital | Ling-Ming TSENG |
| Taiwan | Chi Mei Medical Center, Liou Ying | Shang-Wen CHEN |
| Taiwan | China Medical University Hospital | Hwei-Chung WANG |
| Taiwan | Taichung Veterans General Hospital | Yang, Youngsen |
| Taiwan | Kaohsiung Medical University Chung-Ho Memorial Hospital | Ming-Feng HOU |
| Taiwan | Tri-Service General Hospital | Ming-Shen DAI |
| Taiwan | National Taiwan University Hospital | Chiun-Sheng HUANG |
| Taiwan | Kaohsiung Veterans General Hospital | Hong-Tai CHANG |
